# Study on the effectiveness and safety of Foldable Capsular Vitreous Body implantation

**DOI:** 10.1186/s12886-019-1268-x

**Published:** 2019-12-18

**Authors:** Xiangyang Zhang, Xuemin Tian, Baike Zhang, Lisa Guo, Xiaodan Li, Yong Jia

**Affiliations:** 1 Xinxiang Medical University, Xinxiang, 453003 Henan Province China; 2 The People’s Liberation Army 988th Hospital (formerly the People’s Liberation Army 153rd Hospital), No. 602 Zhengshang Road, Zhengzhou, 450000 Henan Province China

**Keywords:** Foldable capsular vitreous body, Efficacy, Safety, Retinal detachment, Vitreoretinal disease, Vitrectomy, FCVB, IOP

## Abstract

**Background:**

Foldable capsular vitreous body (FCVB) was designed to treat severe retinal detachment. The aim of this study was to evaluate the efficacy and safety of the implantation of foldable capsular vitreous body in 1-year follow-up.

**Methods:**

A retrospective analysis was conducted for 20 patients with severe ocular trauma or silicone oil (SO) dependent eyes underwent vitrectomy and FCVB implantation in a 1-year follow-up. All treated eyes were peformed clinical examinations involved the visual acuity (VA) examination, Goldmann applanation tonometer, noncontact specular microscopy, fundus photography, B-Scan examination and optical coherence tomography (OCT). The groups were compared with t-test and the McNemar - Bowker test.

**Results:**

In 1-year follow-up, 20 eyes were evaluated in the study. FCVB well supported the vitreous retina in all treated eyes, and 6 treated eyes achieved retinal reattachment 12 months after FCVB implantation. There were no significant differences in VA before and after FCVB implantation (*P* = 1.000). In addition, the postoperative IOP markedly elevated from the preoperative IOP of 12.90 ± 7.06 mmHg to 15.15 ± 3.36 mmHg (*P* = 0.000017). The intraocular pressure (IOP) of 10 eyes maintained at a normal level after surgeries. The other 10 eyes showed slightly lower IOP within the acceptable level. Though two patients developed keratopathy and ocular inflammation respectively, other treated eyes were symmetric with fellow eyes showing satisfactory appearance. Moreover, there was no SO emulsification or leakage happened in the observation.

**Conclusions:**

FCVB implantation was an effective and safe treatment in the eyes with severe retinal detachment.

## Background

Over 80% of the structure of the eyeball is natural vitreous body, which contains only 1% of proteins, hyaluronan, lipids and inorganic chemical compounds. Natural vitreous body, which was proved to maintain a certain spatial relationship with dipolar water molecules, has the ability to support the retina, supply nutrition, stabilize intraocular metabolism, act as cell barrier and ocular refractive media, etc [1] However, the composition of natural vitreous body is unclear, which means it is impossible to create artificial vitreous body [2–4]. Furthermore, due to the natural vitreous body is non-renewable [5–7], vitrectomy and SO tamponade are required when vitreoretinal diseases occur, such as retinal detachment caused by severe ocular trauma, post-trauma proliferative vitreoretinopathy, proliferative diabetic retinopathy and endophthalmitis [8–11]. SO is a kind of the vitreous substitutes which also include gases, heavy water, heavy SO. And Vitreous tamponade aims to replace the natural vitreous body with vitreous substitutes to support the retina, promote the anatomical reduction of the retina and prevent the eyeball from shrinking [5, 12]. But current discovered vitreous substitutes have short-term life-span and high toxicity causing severe complications in the eyes [13, 14]. Specially, SO has been proved to be toxic for ciliary body and attribute to emulsificate in the eyeball, though SO has the most stable physical and chemical attributions among all the vitreous substitutes [15]. Researchers have been working hard to find suitable vitreous substitutes in the past decades before the invention of FCVB [12, 16].

FCVB is an innovative product initially developed independently by China [17]. It was designed to refine the way that SO work in the inner cavity of eyeball, and help to support the retina. Since FCVB has excellent mechanical strength, refractivity and biocompatibility, it is able to prevent SO from displacement and emulsification [18]. Moreover, the post-surgical complications are effectively reduced [19]. Unlike SO tamponade, there is no more need for patients to keep prone positions after surgeries [20].

FCVB have been approved in vitro and animal experiments [21–23]. Nevertheless, the clinical safety and efficacy of FCVB have not been verified yet, and previous associated clinical studies were not enough [18, 19, 24, 25]. In this study, FCVB implantation, as a new method treating severe ocular trauma or SO-dependent eyes, was performed on 20 patients with retinal detachment. It aimed to study the clinical safety and efficacy of FCVB for the patients.

## Methods

### Study design

A retrospective study of 20 cases involving 20 patients with FCVB implantation was conducted between September 2017 and September 2018. The basic demographic information of the patients is shown in Table 1. The average age was 40.05 years old (2–65 years old), including 17 males and 3 females. There were 13 cases of eyeball rupture, 5 cases of SO dependent eyes, one case of endophthalmitis, and one case of eyeball atrophy. The study protocol was reviewed and approved by Medical Ethics Committee of the People’s Liberation Army’s 988rd Central Hospital (2017006). The clinical trials strictly adhered to the principles of The World Medical Association Declaration of Helsinki and have been successfully registered.

**Table 1:** Preoperative general information and eye diagnosis information of patients

Patient	Sex	Diagnosis		Initial VA (logMar)	Initial IOP (mmHg)	The time from diagnosis to operation (days)	Final VA (logMar)	Final IOP (mmHg)	Surgical sucess (yes/no)	Follow-up time (months)
		Main diagnosis	other ophthalmological diagnosis							
01	male	1. ocular rupture; 2. vitreous hemorrhage	1.irideremia; 2.lack of lens	NLP	Tn-2	4	NLP	Tn	yes	12
02	male	1. retinal detachment	1. vitreous hemorrhage; 2. anterior chamber hemorrhage;3. irideremia; 4. lack of lens	NLP	Tn + 2	3	LP	Tn-1	yes	12
03	male	1. ocular rupture; 2. retinal detachment	1. eyelid trauma	NLP	Tn-2	8	NLP	Tn	yes	12
04	female	1 ocular rupture; 2. retinal detachment	1. irideremia; 2. lack of lens	NLP	Tn-1	28	LP	Tn	yes	12
05	male	1. ocular rupture; 2. retinal detachment	1. iridodialysis	NLP	Tn-1	9	NLP	Tn	yes	12
06	male	1. ocular atrophy after SO tamponade	1. endophthalmitis; 2. corneal degeneration; 3. lack of lens	NLP	Tn-1	16	NLP	Tn	yes	12
07	male	1. retinal detachment; 2. silicone oil dependent eye	1. optic atrophy; 2. irideremia; 3. lack of lens	HM	Tn-1	15	HM	Tn	yes	12
08	male	1. ocular atrophy	1. exotropia; cataract; 2. lens dislocation	NLP	Tn-1	6	NLP	Tn	yes	12
09	male	1. ocular rupture; 2. retinal detachment;3. chorioretinal detachment	1. ocular rupture	HM	Tn-1	4	HM	Tn-1	yes	12
10	male	1. recurrent retinal detachment; 2. ocular atrophy	1. lack of lens	FC/30 cm	Tn-1	2	HM	Tn-1	yes	12
11	male	1. recurrent retinal detachment	1. lack of lens; 2. secondary glaucoma	HM	Tn + 1	2	LP	Tn-1	yes	12
12	female	1. retinal detachment	1. intraocular lens implantation; 2. exotropia; 3. vitreous opacities	NLP	Tn-1	5	NLP	Tn	yes	12
13	male	1. ocular rupture		LP	Tn	14	LP	Tn-1	yes	12
14	male	1. ocular rupture; retinal detachment; 2. choroidal detachment	1. vitreous hemorrhage	NLP	Tn-1	14	NLP	Tn	yes	12
15	male	1. retinal detachment; 2. ocular atrophy	1. traumatic cataract, 2. vitreous opacity, exotropia	NLP	Tn-1	2	NLP	Tn-1	yes	12
16	male	1. SO dependent eye	1. high myopia;2. corneal degeneration	LP	Tn-1	6	LP	Tn-2	yes	12
17	male	1. recurrent retinal detachment;2. intraocular lens implantation	1. irideremia; 2. lack of lens	HM	Tn-1	4	LP	Tn-2	yes	12
18	female	1. ocular rupture; 2. retinal detachment	1. irideremia; 2. lack of lens	LP	Tn-1	0	HM	Tn-1	yes	12
19	male	1. ocular rupture; 2. retinal detachment		LP	Tn-2	20	LP	Tn	yes	12
20	male	1. ocular rupture; 2. retinal detachment	1. anterior chamber hemorrhage; 2. vitreous hemorrhage	NLP	Tn-1	6	NLP	Tn-1	yes	12

The age - ranges of patients: 2–65 years. Visual acuity (VA): NLP – No light perception; LP – Light perception; HM – Hand move; FC – Finger count. Intraocular pressure (IOP): Tn-2, 5–9 mmHg; Tn-1, 10–14 mmHg; Tn, 15–20 mmHg; Tn + 1, 25–34 mmHg; Tn + 2, 35–50 mmHg

### Inclusion criteria

Patients with a severe retinal detachment that could not be cured with SO tamponade, such as posterior scleral ruptures with large disruptions of the retina or severe scleral ruptures with retinal detachments and choroidal damage, or they had to have rigid retinal re-detachments or inferior holes that occurred after more than 3 months of SO tamponade. In addition, the treated eyes should below PVR-B, its axial length ≤ 26 mm. A third-party committee of ophthalmologists should confirm that the detached retina could not be reattached with SO tamponade and satisfied the requirements of FCVB implantation.

If the retinal detachment could be treated with SO tamponade, the surgeon could stop the procedure and deny FCVB implantation.

### Excluded criteria

The exclusion criteria were contraindications approved by the China Food and Drug Administration (CFDA). Excluded criteria were scar physique, glaucoma, eye inflammation, macular diseases and other diseases that the patients were deemed unsuitable for FCVB implantation. Adverse events should be recorded, such as unbearable foreign body sensations, uncontrollable bleeding, severe inflammation, endophthalmitis, severe disturbance of eye movement, diplopia and sympathetic ophthalmia during the study.

### Surgical technique

After Inclusion, 20 patients (20 eyes) underwent FCVB implantation. The operating procedures consist of 9 steps: (1) Sterilization and retrobulbar anesthesia was performed before operation. (2) A three-ports vitrectomy was conducted to remove the intraocular lesion tissue of the retina and hemorrhage. (3) After conducting paracentesis of the anterior chamber, a 3.5 mm incision was create on the sclera as FCVB implantation site at either the 4 or 8 o’clock position 4.5 mm away from the corneal limbus. Perform gas-fluid exchange to lift the IOP over 50 mmHg and evaluate the state of retina with fundus observation. (4) The air tightness of FCVB was tested under ddH_2_O. (5) FCVB was properly fold and implanted into the cavity of eyeball through the incision with push injector. The lens surface of the capsule of FCVB should be placed facing the lens. If there is tilting, with iris repositor, the position of FCVB capsule can be adjusted properly. (6) The incisions were sutured carefully with an 8–0 absorbable suture. Then the SO was slowly injected into the capsule through the drainage valve with syringe until the IOP is approximately 15 mmHg. The injected SO volume, which should be less than the maximum injection volume of each model, was recorded. (7) The anterior chamber was fulfilled with adequate volume of viscoelastic. (8) Postoperative observation (capsule’s expansion, air bubble and the blood supply of retina in the capsule) to the end of the drain tube by connecting to the optical fibers. (9) The drain tube was ligated with the 5–0 non-absorbable suture and affix it to the sclera.

Postoperative medications: Methylprednisolone 80 mg intravenous drip for three days, then prednisone 70 mg, 10 mg per week reduction. If for the aged with systemic diseases, such medication should be used cautiously.

### Follow-up examinations

Ocular examinations were performed at baseline, 1, 2, 3, 6 and 12 months after FCVB implantation. The arrangements of ocular examinations consist of VA with Snellen eye charts (transformed to logMAR) and IOP with Goldmann applanation tonometry (GAT; Haag-Streit AG, Köniz, Switzerlan), traditional slit lamp biomicroscopy (Nikon FS-2, Nikon Inc., Melville, NY), direct ophthalmoscopy, and fundus photography (TRC- 50EX; Topcon, Tokyo, Japan). A-scan ultrasound (CineScan A/B; Quantel Medical, Bozeman, MT), B-scan ultrasound (CineScan A/B; Quantel Medical, Bozeman, MT), noncontact specular microscopy (SP-3000P; Topcon, Tokyo, Japan), optical coherence tomography (OCT, Visante; Carl Zeiss Meditec, Dublin, CA), ultrasound biomicroscopy (UBM, SW-3200 Kinscan; Suoer, Tianjin, China) and were performed at baseline and 3 months after implantation.

### Outcome measures

The primary outcome measure was the rate of complete retinal reattachment detected by B-scan ultrasound after the FCVB implantation. If conditional, OCT was also conducted.

The secondary outcome measure included VA, IOP, and axial length, as determined by the Snellen chart, Goldmann applanation tonometry, and A-scan, respectively. Classification of VA follows the typical scoring system: no light perception represented 0; light perception represented 1; hand motion represented 2; finger the perception represented 3; ≥0.05 acuity represented 4; and ≥ 0.1 acuity represented 5. The preoperative VA before FCVB implantation were at relative low level, representing less than 2.

All outcomes derived from photograph were determined by independent grading of retinal photographs, performed by the third-party Fundus Photograph Reading committee which consisted of ophthalmologists.

### Statistical analysis

In this study, SPSS 15.0 (SPSS Inc., Chicago, IL, USA) was used for data analyses. IOP are expressed as Mean ± SD. A paired-samples test was used for comparison of IOP between baseline and 6 months after surgery. Analysis of categorical data followed by the McNemar - Bowker test was used to analyze between-group differences of VA, as well as IOP, and Fisher’s exact test was used when expected cell counts were below five. A two-tailed value of *P* < 0.05 was considered statistically significant.

## Results

### Baseline information

Twenty patients (20 eyes) were enrolled in the study between September 2017 and September 2018. The demographic and ocular condition of patients at baseline examination are shown in Table 1. The mean age of the patients was 40.05 ± 20.66 years, and 85% of patients were male. Case 9 and Case 12 had severe ocular rupture with retinal and choroidal detachments. All the patients suffered severe ocular trauma or silicone oil dependent eye. Since that, the patients were performed vitrectomy and FCVB implantation. Also, all the patients had signed informed consent forms.

<Table 1 Preoperative general information and eye diagnosis information of patients>

### Analysis of the efficacy of FCVB implantation

Contrast analysis of VA and IOP before and after the operation was conducted in 20 patients. Before operation, the VA were unsatisfactory, 55% of the patients under NLP condition. Only one patient’s VA had an regression from finger count in 30 m scopes (FC/30 cm) to light perception or hand moving (LP-HM), while there were no significant changes in the VA of the other patients before and after the operation (*P* = 1.000), as seen from Table 2. There was obvious improvement of IOP after FCVB surgery. Contrast to the preoperative IOP was 12.90 ± 7.06 mmHg, the postoperative IOP elevated to 15.15 ± 3.36 mmHg, as seen from Table 2. According to the results of IOP records, it was normal in 1 cases, low in 17 cases and high in 2 cases before operation, while 10 cases showed low IOPs, and the IOP of other 10 cases returned to normal level in 12 months after the operation. The difference between preoperative and postoperative IOPs was statistically significant (*P* = 0.000017). The results are shown in Table 3.

<Table 2 Preoperative and Postoperative VA (Number of Eyes)>

<Table 3 Preoperative and Postoperative Changes in IOP (Number of Eyes)>

Twelve months after FCVB implantation, B-ultrasound was conducted and 6 of the 20 patients developed retinal reattachment, while the retina of the other 14 was lost or damaged, due to severe eye damage. With B-ultrasound, the novel FCVB was found properly positioned and was well distributed within the vitreous cavity. Moreover, no severe complications associated with FCVB were detected, though some patients suffered mild hemorrhage and omental proliferation caused by previous severe ocular damage. Two cases, which had complicated conditions, are detailed in case 3 and case 12.

**Table 2:** Results

	Visual Acuity Analysis (*n* = 20)			IOP (mmHg)
	NLP	LP-HM	FC/30 cm	
Preoperation	11	8	1	12.55 ± 7.51
Postoperation	9	11	0	14.36 ± 3.85

The total number of treated eyes (n) = 20; Visual acuity analysis: *NLP* No light perception, *LP-HM* Light perception or hand move, *FC*/30 cm Finger count, range within 30 cm

**Table 3:** Preoperative and Postoperative Changes in Intraocular Pressure

Intraocular Pressure	Tn-2	Tn-1	Tn	Tn + 1	Tn + 2
Preoperation	3	14	1	1	1
Postoperation	2	8	10	0	0

The total number of treated eyes = 20; Follow-up period: 12 months (September 2017–September 2018);

Tn: Estimation of IOP by finger

Tn-2, 5–9 mmHg; Tn-1, 10–14 mmHg; Tn, 15–20 mmHg; Tn + 1, 25–34 mmHg; Tn + 2, 35–50 mmHg;

*Indicates statistical significance (*P* < 0.05).postoperative IOP compared to preoperative IOP

**Fig. 1:**
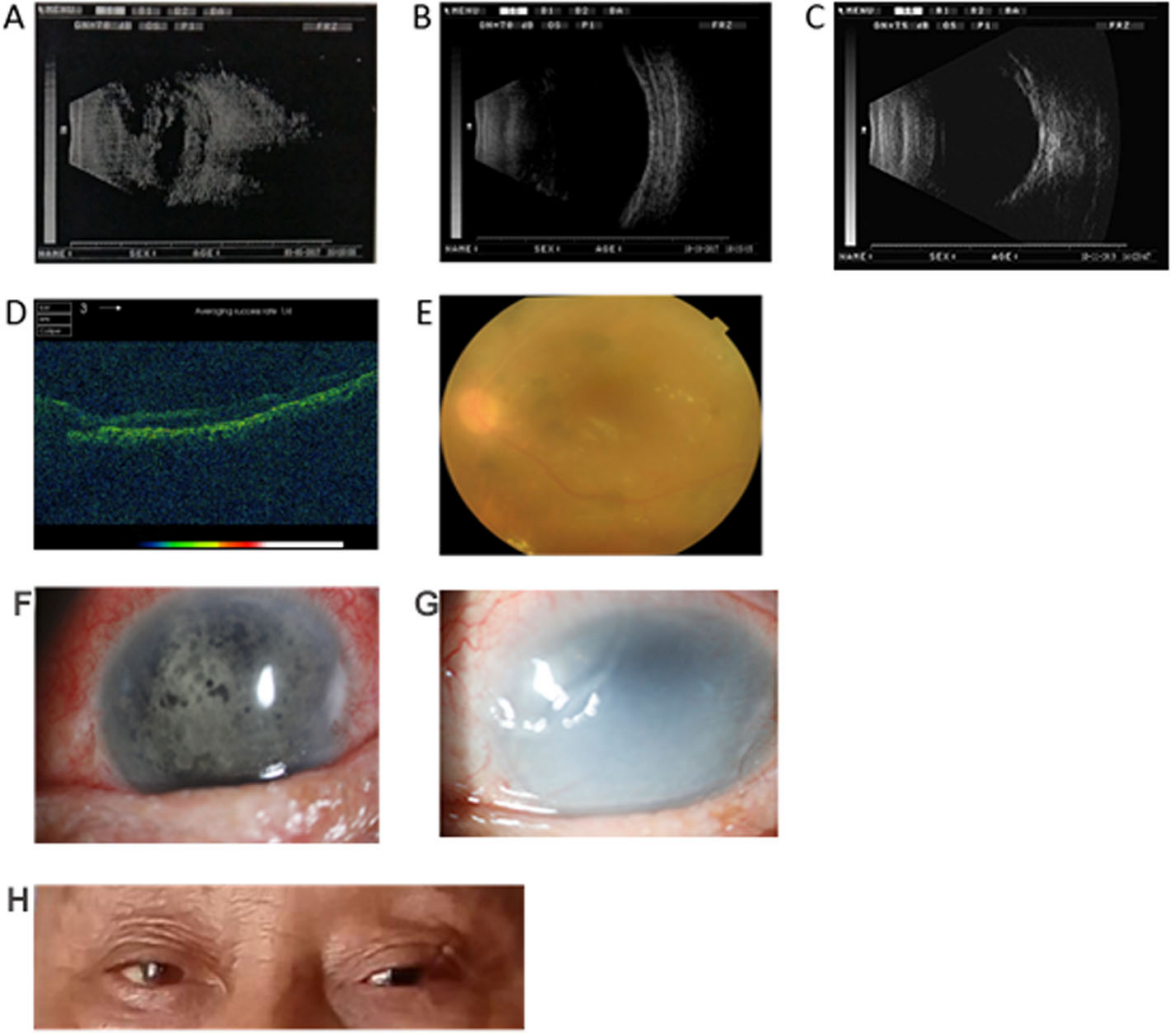
Postoperative follow-up results of FCVB implantation in patient 3. (**a**) Preoperative B-ultrasound. (**b**) B-ultrasound 6 months after operation. (**c**) B-ultrasound 12 months after operation. (**d**) OCT 6 months after operation. (**e**) Fundus photography 6 months after operation. (**f**) Slit lamp biomicroscopy 6 months after operation. (**g**) Slit lamp biomicroscopy 12 months after operation. (**h**) Binocular appearance 6 months after operation

Case 3 - The preoperative B-ultrasound showed strong echogenic spots (bleeding) visible in the vitreous cavity connected to the wall of the ball (Fig. 1a). The cornea of case 3 was transparent, the anterior chamber was clear, the FCVB position was positive, a layer of mechanical membrane was seen on the anterior surface and the IOP was 10 mmHg six months after operation (Fig. 1b). Twelve months after operation, the retina owned firm connection to the inner scleral wall (Fig. 1c). Moreover, The OCT showed the normal fovea’s structure and well recovered retina (Fig. 1d). The postoperative B-ultrasounds of cases 3 indicated that there was pseudo-expansion of the eyeball, while the FCVB well spread in the vitreous cavity without any obvious abnormalities (Fig. 1b). Examination of the fundus showed that the fundus was clearly visible after the FCVB was filled and adequately supported the retina. The retina was flat without wrinkles during the 12 - month post - implantation period (Fig. 1e).

**Fig. 2:**
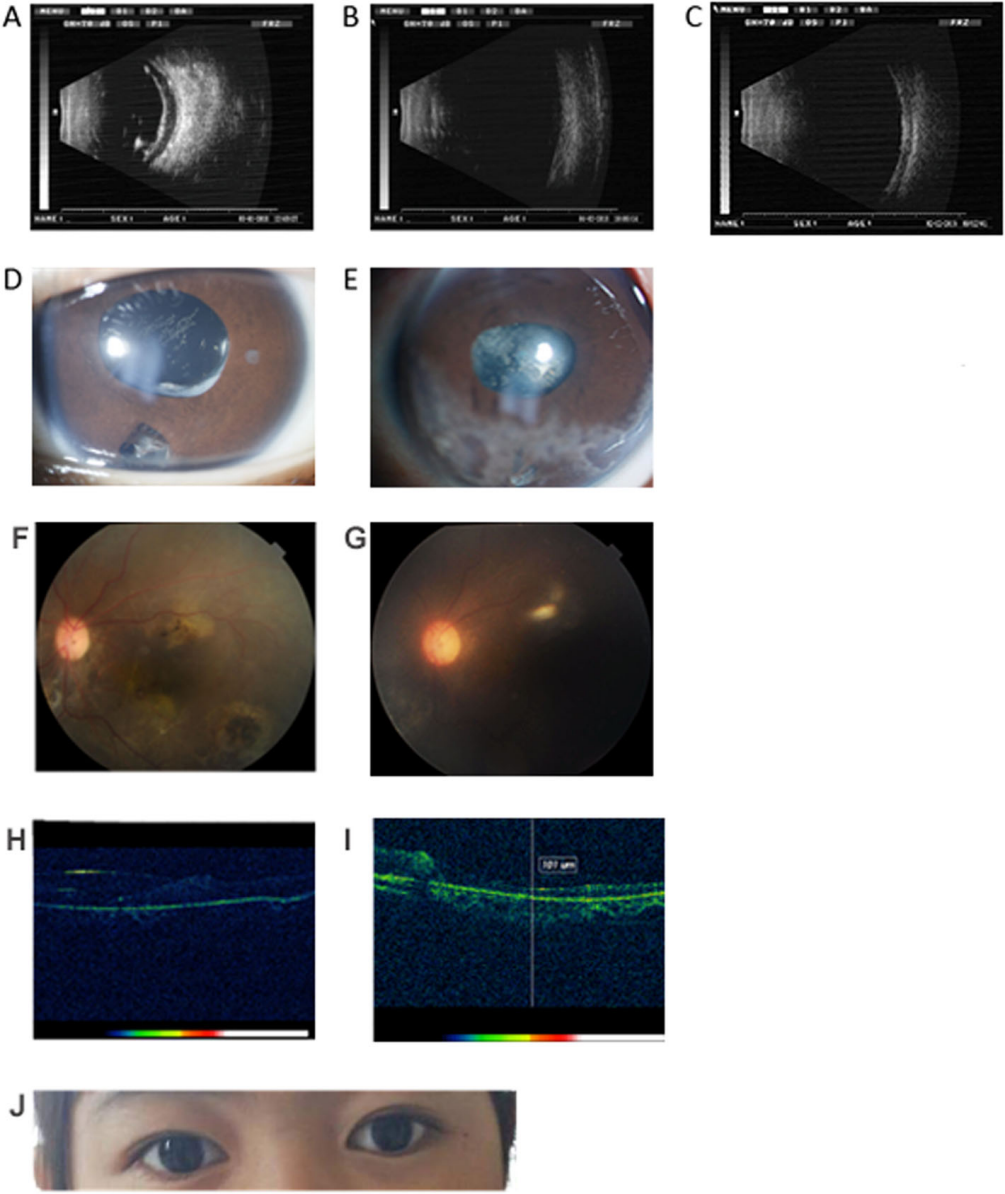
Postoperative follow-up results of FCVB implantation in patient 12. (**a**) Preoperative B-ultrasound. (**b**) B-ultrasound 6 months after operation. (**c**) B-ultrasound 12 months after operation. (**d**) Slit lamp biomicroscopy 6 months after operation. (**e**) Slit lamp biomicroscopy 12 months after operation. (**f**) Fundus photography 6 months after operation. (**g**) Fundus photography 12 months after operation. (**h**) OCT 6 months after operation. (**i**) OCT 12 months after operation. (**j**) Binocular appearance 6 months after operation

Case 12 - The preoperative B-ultrasound showed visible banded echoed in the vitreous body and visible point sheet turbidity between the walls of the eyeball (Fig. 2a). The cornea of case 12 was transparent, the anterior chamber was clear, the FCVB position corresponded to the standards and the IOP was 13 mmHg after 6 months (Fig. 2d). At 12 months after operation, there is minor corneal degeneration happened, while it did not have great influence on the visual acuity (Fig. 2e). The OCT result indicated that the fovea had a vague structure and that there were no obvious abnormal signs on the retina (Fig. 2h and Fig. 2i). The postoperative B-ultrasounds of cases 12 also showed well support of the FCVB for retina in the vitreous cavity without any obvious abnormalities (Fig. 2b). In the examination of the fundus, the clearly visible fundus was observed, which was consistent with the OCT and B-ultrasounds results. Besides, fulfilled FCVB solidly supported the retina and the entire eyeball. There were no obvious wrinkles seen on the well-recovered retina during 12 - month postoperative period (Fig. 2f and Fig. 2g).

Both in cases 3 and case 12, the eyes were symmetrical and the eyeballs were normal after 6 months (Fig. 1h, Fig. 2j).

According to these data, the FCVB well distributed in the vitreous cavity and that the support for the retina was uniform.

### Safety analysis of FCVB implantation

All 20 patients had successful FCVB implantation combined with pars plana vitrectomy. And there were no intraoperative complications. However, 3 patients presented a slight degree of haematocele in the anterior chamber after the operation. The presumed reason for this complication was that it may be hemorrhage of the incision, or the tip of the product may have pierced the intraocular tissue during implantation. The hemorrhage of the anterior chamber disappeared after conservative treatments such as haemostasis and lowering of the IOP. Another patient developed an inflammatory reaction in the eye after the operation but the condition began to stabilize after anti-inflammatory and hormone therapy. In addition, one patient developed eyeball atrophy after operation, due to the mistakes of choosing the models of FCVB. The condition was stable and the patients were satisfied after subsequent adjustments. The remaining patients did not have obvious complications, such as anterior chamber hemorrhage, development of a fibrous membrane, exposure of products, capsule rupture, SO - spillage and high IOP.

## Discussion

Precisely, there is no perfect material that has the ability to completely mimic the functions of natural vitreous body along [16]. Basically, the vitreous substitutes can be divided into two groups: liquid and gas. Both SF_6_ and C_3_F_8_, which belongs to gas, are the common vitreous substitutes. But they have the same defects that they tend to be absorbed and their volume will expand if the patients are at high altitude. Compared to gas, SO, as a kind of liquids, has more relatively physical stability. Nevertheless, SO owns two major defects which lacked an effective solution. On the one hand, directly contacted to the tissue of inner eyeball, emulsification of SO was inevitable [26]; On the other hand, more and more studies have indicated the biological toxicity of SO. Especially for ciliary body, after long time exposure to SO, it will lose its basic function and disable of secreting aqueous humor [27, 28]. Therefore, the failure of aqueous humor circulation was triggered and treated eyes will facing enucleation or evisceration in the future [29, 30].

FCVB was designed to support the retina combined with more than 5000 cts SO injected through the drain tube, which is a brand-new therapy strategy. FCVB implantation had the aid of the surface tension of SO, not only 360° supported the retina, but also isolated the SO with the capsule of FCVB. By extracting or supplying SO through the drainage valve of the FCVB, the IOP can be regulated [31]. Since that, the key problem of simply SO tamponade was solved, no more emulsification of SO would occur under the protection of FCVB. Furthermore, patients were no needs to keep prone posture after surgery.

Made from medical grade biocompatible polymer (FDA registered material), the FCVB can directly contact with the tissue in the eyeball and barely induce irritation. Moreover, in the rabbit model of PVR, SO-injected FCVB closely fitted the inner structure of eyeballs and restored basic features (support, refraction and cellular barriers) without causing any complication after implantation. In addition, in vitro studies, no significant changes of genes or proteins of retinal pigment epithelium were detected after FCVB was implanted into eyeball [32–35]. Associated results are the proof that FCVB owned ideal biocompatibility, but still need further exploration by clinical trials [18]. It is noticed that, if severe infection happens, FCVB can be removed easily from a 3.0 mm – 4.0 mm incision.

In this study, 20 patients requiring vitrectomy were subjected to pars plana vitrectomy and FCVB implantation. The ratio of retinal reattachment was a key metric of therapy effect of FCVB surgery treating retinal detachment, determined by fundus photography, B-Scan examination and OCT. And the results showed that all the treated eyes achieved successful anatomical reduction after FCVB implantation, which indicated the excellent efficacy of FCVB. Besides, IOP, which was monitored with Goldmann applanation tonometer in the follow-up, returned to normal level after surgery. But there was little difference between preoperative and postoperative VA. Moreover, no severe ocular atrophy happened.

Consistent to its design theory, by isolating the SO from anterior chamber, posterior chamber and other intraocular tissues, FCVB effectively prevented SO from emulsification, dramatically reduced corneal degeneration, high IOP and other complications. According to the records, only two patients had adverse events; one had mild eyeball atrophy and the other had an inflammatory reaction, which improved and stabilized after treatment. The feedback from the latter patient was satisfactory. Therefore, associated data further confirmed the safety of FCVB implantation. FCVB showed ideal application value in clinic.

It was noticed that the prognosis for children after the operation, compared to adult with severe eye diseases, was worse, and the consequences are more serious [25]. An integral eyeball is a vital part of human appearance. And appearance has great impact on the development of children. The patients with severe retinal detachment always have undesirable appearance, which severely impacted their psychological state. At present, vitrectomy is an indispensable therapy for severe ocular trauma in children, as well as avoids aesthetic defects caused by eyeball removal [11]. In this study, three children were enrolled in the study and their IOP returned to normal without the occurrence of any postoperative complications and adverse reactions after surgery. The aesthetics of the children after vitrectomy and FCVB implantation was satisfactory.

Given above results, FCVB was proved to be an ideal vitreous substitute, could restore the structure and basic functions of eyeballs, as well as maintain good aesthetic.

Beyond that, since there are numerous 300 nm tiny apertures designed on the surface of the capsule, the FCVB can act as an intravitreal drug delivery system (DDS), and release drugs following associated pharmacokinetics synchronously. Plus, FCVB will not change the chemical property of drugs [34, 36–38]. For instance, the implanted FCVB can sustainably and mechanically release dexamethasone sodium phosphate (the molecular mass = 516.41 Da) through the apertures in a time-dependent and a dose-dependent manner [22]. Hence, the FCVB is a new potential approach to meet demand of combining vitreous substitutes and drug treatments. As the clinical data showed, FCVB implantation is potential to be an effective and safe technique for the eye in severe traumatic and infectious disease. But this study was not comparative study and lack of long-term follow-up. To ensure the superiority and safety of FCVB among all the vitreous substitutes, it is certainly worth conducting large-scale comparative clinical trials in the future.

## Conclusions

The findings presented that FCVB implantation can be a new and effect method for the patients with severe retinal detachment or silicone oil - dependent eyes. In the prognosis, FCVB can support the retina, maintain the eyeball shape and stabilize the Visual acuity. In the meanwhile, the SO in FCVB was able to be protected from emulsification. So, there is bright prospects of FCVB in clinical application.

## Data Availability

All the data supporting our findings is contained within the manuscript and tables. The relevant raw data will be freely available from the People’s Liberation Army’s 988rd Central Hospital by request.

## Abbreviations

CFDA: China food and drug administration
DDS: Drug delivery systems
FC: Finger count
FCVB: Foldable capsular vitreous body
HM: Hand motion
LP: Light perception
OCT: Optical coherence tomography
SPSS: Statistical product and service solutions
